# *QuickStats:* Infant Mortality Rates for Metropolitan and Nonmetropolitan Counties,* by Single Race and Hispanic Origin — National Vital Statistics System, United States, 2019

**DOI:** 10.15585/mmwr.mm7044a4

**Published:** 2021-11-05

**Figure:**
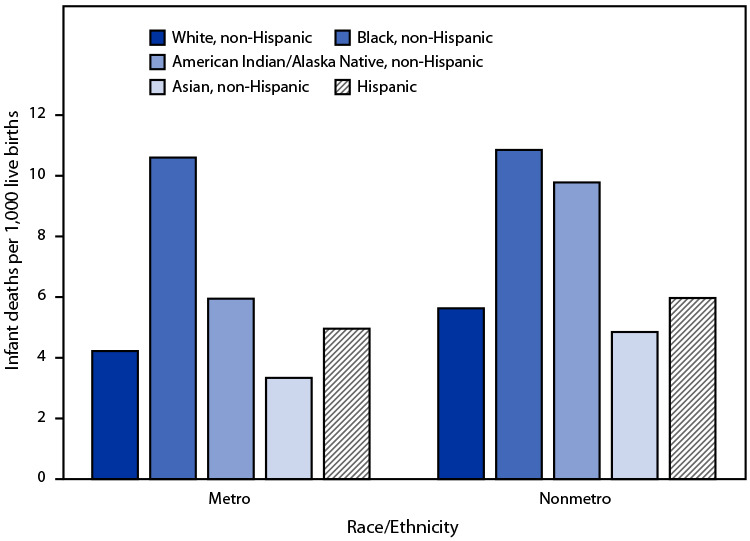
In metropolitan counties, infant mortality rates were highest for infants of non-Hispanic Black mothers (10.60 infant deaths per 1,000 live births), followed by infants of non-Hispanic American Indian or Alaska Native (5.95), Hispanic (4.96), non-Hispanic White (4.22), and non-Hispanic Asian (3.34) mothers. In nonmetropolitan counties, the mortality rate was also highest for infants of non-Hispanic Black mothers (10.85), followed by infants of non-Hispanic American Indian or Alaska Native (9.78), Hispanic (5.97), non-Hispanic White (5.63), and non-Hispanic Asian (4.85) mothers. The infant mortality rate was significantly lower for infants of non-Hispanic White, non-Hispanic American Indian or Alaska Native, and Hispanic mothers in metropolitan counties compared with nonmetropolitan counties; differences in rates between metropolitan and nonmetropolitan counties for infants of non-Hispanic Black and non-Hispanic Asian mothers were not statistically significant.

**Source:** National Vital Statistics System. Linked Birth and Infant Death Data. https://www.cdc.gov/nchs/nvss/linked-birth.htm

